# *In vitro* characterization of the adhesive factors of selected probiotics to Caco-2 epithelium cell line

**DOI:** 10.1080/13102818.2014.969948

**Published:** 2014-10-29

**Authors:** Zhechko Dimitrov, Irina Gotova, Elena Chorbadjiyska

**Affiliations:** ^a^ R&D Center, LB-Bulgaricum PLC., Sofia, Bulgaria

**Keywords:** probiotics, adhesion, cell-wall proteins

## Abstract

The aim of the present study was to analyse the type of adhesive factors of selected probiotic strains. A large number of lactic acid bacteria with intestinal and dairy origin were collected and assessed for adhesion on Caco-2 cell line. From the best adherent bacteria, four strains were selected for further research: *Lactobacillus gasseri* G7, *L. plantarum* F1, *L. helveticus* AC and *L. delbrueckii* subsp*. bulgaricus* B14. The average number of adhered bacteria was 17 per one Caco-2 cell in the case of *L. gasseri* G7 and 21 per cell in the case of *L. plantarum* F1. Treatment with ethylenediaminetetraacetic acid (EDTA), trypsin and metaperiodic acid in separate assays revealed that cell-bonded extracellular proteins were responsible for the adhesion of the selected *L. gasseri*, *L. plantarum* and *L. helveticus* strains, in contrast to the *L. delbrueckii* subsp*. bulgaricus* strain, whose adhesive factors were identified as cell-bonded exopolysaccharides. The cell-wall proteins from the first three strains were isolated, fractionated and assessed for adhesion to Caco-2 cells. Based on the attachment properties of the purified proteins towards Caco-2 cells, it was clearly proved exactly which proteins are involved in the adherence. *L. plantarum* F1 strain contains two adhesive proteins in contrast to the other selected strains containing one adhesive protein each. The determination of the factors mediating the adhesive abilities of the selected strains provides important information about the possible ways to preserve and increase adhesive properties towards epithelium cells.

## Introduction

Probiotics are viable bacteria that have beneficial effects on the health of the host.[1] Many probiotic bacteria are lactic acid bacteria and are useful in the treatment of disfunctions in the intestinal microflora and abnormal gut permeability.[2,3] Proposed mechanisms through which the ingested probiotics may subsequently benefit their host include the production of antimicrobial factors, competition for nutrients, degradation of toxins and immune modulation.[4] However, of the main criteria for selecting probiotic strains, adherence to intestinal epithelia is thought to be paramount.[5,6] Indeed, adhesion to epithelial cells and/or mucus appears to mediate colonization of the gastrointestinal tract by lactobacilli and may be a prerequisite for competitive exclusion of enteropathogenic bacteria [7] and immune modulation of the host.[8–10] Successful probiotic bacteria are usually able to colonize the intestine, at least temporarily, by adhering to the intestinal mucosa.[11,12] Studies have also suggested that adhesive probiotic bacteria could prevent the attachment of pathogens, such as *Salmonella* and *Clostridia*, and stimulate their removal from the infected intestinal tract.[3,13] *In vitro* tests for comparative evaluation of the adhesive ability of the strain *Lactobacillus plantarum* AC131 on HT-29 and HeLa cell lines have been carried out.[14] Laboratory models using human intestinal cell lines such as Caco-2 [15,16] and HT-29 [17] have been developed to study the adhesion of probiotic lactic acid bacteria and their competitive exclusion of pathogenic bacteria.[3]

The aim of this study was to assay of the adherence of lactobacilli with intestinal and dairy origin to human epithelial cells, using a quantitative approach, and to select strains with proved adhesion properties on human epithelium cell line Caco-2 as well as to identify the nature of adhesive compounds. This approach provides a better insight into the mechanism of adherence of probiotic bacteria, and thus allows development of more efficient probiotic products.

## Materials and methods

### Bacterial strains

Intestinal and dairy lactobacilli were isolated after plating of 0.1 mL of respective dilutions of faecal or milk/cheese homogenates on Man–Rogosa–Sharpe (MRS) agar (Merck, Darmstadt, Germany). The plates were incubated at 37 °C for three days under anaerobic conditions (10% CO_2_, 80% N_2_, 10% H_2_). The single colonies were purified thrice and the species belonging and strain identity were determined by species-specific polymerase chain reaction (PCR), amplified ribosomal DNA restriction analysis, sequencing of 16S rDNA, and pulsed field gel electrophoresis.[18]

### Preparation of the cell lines for the quantitative assessment of the adhesion

The intestinal cell culture Caco-2 was used in the adhesion assay. This human colon adenocarcinoma cell line was obtained from the American Type Culture Collection. The cells were cultured in Dulbecco's modified Eagle's minimal essential medium (DMEM, GIBCO-BRL), containing 25 mmol/L of glucose, 20% (vol/vol) of heated inactivated foetal calf serum (GIBCO-BRL), and 1% non-essential amino acids (GIBCO-BRL). The cells were grown at 37 °C in 5% CO_2_. At approximately 95% confluence, the monolayers were detached by incubating with a 0.25% trypsin solution (Gibco) for 5 min at 37 °C. For the adhesion assay, monolayers of Caco-2 cells were prepared in two-chamber slides (Lab-Tek chamber slide; Nunc Inc.) by inoculating 2.8 × 10^5^ viable cells into 2 mL of culture medium. The medium was replaced every two days. Fifteen-day post-confluent Caco-2 monolayers were washed three times before the adhesion assay with 1 mL of sterile phosphate-buffered saline (PBS).

### Preparation of bacterial suspensions for quantitative assessment of adhesion

The strains *L. plantarum* F1, *L. gasseri* G7, *L. helveticus* AC and *L. delbrueckii* subsp. *bulgaricus* B44, which demonstrated the best adhesion properties, were cultured in MRS broth (Oxoid) at 37 °C for 18 h before adherence assays were performed. Bacterial cells and spent culture supernatant were separated by centrifugation at 3000 × *g* for 10 min. The bacterial cells were washed twice in quarter-strength Ringer's solution and resuspended in DMEM to a concentration of 1 × 10^8^ CFU/mL.

### Quantitative assessment of the adhesion

One millilitre of the test bacteria was added to 1 mL of complete Caco-2 medium. This suspension (2 mL) was added to each chamber of the two-chamber slide and incubated at 37 °C, in a 5% CO_2_–95% air atmosphere, with gentle rocking. After incubation for 60 min the monolayers were washed five times with sterile PBS (pH 7.2) and fixed with methanol, Gram stained, and examined microscopically. Each adherence assay was conducted in triplicate, and the number of adherent bacteria was counted on about 1000 Caco-2 cells, in 60 randomly selected microscopic fields. To simulate the physiological pH condition of the gastrointestinal tract, all experiments were done at pH 7.

### Evaluation of the nature of the adhesive factors

To evaluate the nature of the adhesive factors, the bacterial cells were treated with trypsin (2.5 mg/mL, Sigma) for 60 min at 37 °C, centrifuged, washed twice in quarter-strength Ringer's solution and resuspended in MRS before the adhesion assay. To determine the involvement of carbohydrates in the adherence properties, bacterial cells were preincubated with metaperiodate (50 mmol/L, Sigma) for 30 min at 37 °C, centrifuged, washed twice and resuspended as before. Alternatively, the Caco-2 monolayers were washed five times with 2 mL of the chelating agent ethylenediaminetetraacetic acid (EDTA, 20 mmol/L, Sigma) in PBS following addition of the bacterial cells.

### Assessment of cell-borne factors with adhesive properties

In order to determine the cell-wall proteins with adhesive properties, the strains *L. plantarum* F1, *L. gasseri* G7, and *L. helveticus* AC were subjected to a procedure for extraction of cell-wall associated proteins.[19] The cell-wall extracts were divided into two parts: the first one was incubated for 2 h onto a Caco-2 monolayer and the second one was a control. The aliquots from the two parts were loaded onto polyacrylamide gel and the electrophoretic process was conducted in the presence of sodium dodecyl sulphate (sodium dodecyl sulphate–polyacrylamide gel electrophoresis, SDS-PAGE) as previously outlined.[19] The protein fragments with reduced relative intensity, compared to the control cell-wall preparation, were extracted from the gel in a preparative scale and were labelled with an Alexa 488 fluorescent dye as per the manufacturer's protocol (Molecular Probes, USA). The purified fluorescent conjugate was resuspended in PBS solution and incubated with the Caco-2 cells for 2 h at 37 °C in a humidified atmosphere of 5% CO_2_. The protein-Alexa 488 fluorescent conjugate PBS solution was removed from the walls of the plate and washed five times with 2 mL of PBS. The results from the adhesion degree of the protein-fluorescent dye conjugates were obtained using a fluorescence microplate reader (Perkin Elmer).

## Results and discussion

One hundred and eighty *Lactobacillus* strains with intestinal and dairy origin were assessed for adhesion on the human epithelial cell line Caco-2. Two intestinal strains *L. plantarum* F1 and *L. gasseri* G7 (21 and 17 bacteria per epithelial cell, respectively) were selected as the best adhesive strains. Of the dairy strains, adhesive properties were demonstrated by *L. helveticus* AC and *L. delbrueckii* subsp*. bulgaricus* B14 (6 and 8 bacteria per epithelial cell, respectively). The results after the adhesion assay presented as adhesive bacteria per average eukaryotic cell are given in Table 1. The results suggest that the strains with the best adhesive properties belong to *L. gasseri* and *L. plantarum* species. Thus, in the present research three better adherent strains were found: one *L. plantarum* and two *L. gasseri*. Among the strains of dairy origin, one *L. delbrueckii* subsp*. bulgaricus* strain and one *L. helveticus* strain demonstrated the best adhesive properties. That is why they were selected for the study of the nature of the adhesive factors together with the best adherent strains *L. gasseri* G7 and *L. plantarum* F1.

**TABLE 1.** Number of adherent bacteria from different species per average eukaryotic cell.

Species	Number of strains	<5 per cell	5–10 per cell	10–15 per cell	>15 per cell
*L. plantarum*	24	17	4	2	1
*L. casei*	26	24	1	1	0
*L. gasseri*	17	10	3	2	2
*L. fermentum*	21	19	2	0	0
*L. acidophilus*	4	3	1	0	0
*L. helveticus*	46	45	1	0	0
*L. delbrueckii* subsp*. bulgaricus*	28	27	1	0	0
*L. rhamnosus*	14	12	2	0	0

**Figure 1.**
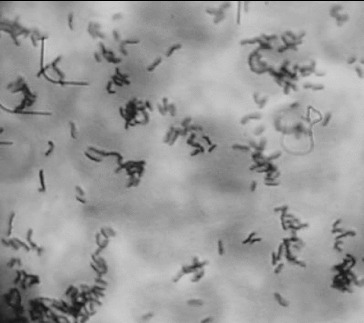
*In vitro* evaluation of the adhesion potential of *L. gasseri* G7 on Caco-2 cell line.

Figure 1 shows the image of a well adherent strain *L. gasseri* G7. The bacterial cells were concentrated selectively onto the eukaryotic cells in contrast to the lack of bacteria between the Caco-2 cells. This proves the specific adhesion to the eukaryotic cells excluding nonspecific interactions between the bacteria and the eukaryotic monolayer. The intensive washing procedures after incubation of the bacteria on the monolayer helped to eliminate the bacterial cells between the eukaryotic cells (Figure 1).

**Figure 2.**
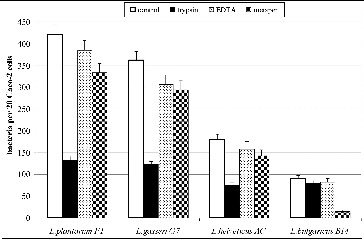
Effect of treatment with trypsin, metaperiodic acid and EDTA on the adhesive properties of *L. plantarum* F1, *L. gasseri* G7, *L. helveticus* AC and *L. delbrueckii* subsp*. bulgaricus* B14. Mean values from three experiments; error bars represent standard deviations.

The treatment of the adhesive strains with trypsin, metaperiodic acid and EDTA revealed that cell-bonded proteins are responsible for the adhesion of the selected *L. gasseri* G7, *L. plantarum* F1 and *L. helveticus* AC strains, in contrast to *L. delbrueckii* subsp*. bulgaricus* B14 strain where cell-bonded polysaccharides were involved in the adhesion. The results from the adhesion assay after the treatment of bacterial strains with different factors are given in Figure 2. After the treatment with trypsin the number of adherent bacterial cells collapsed in the case of strains *L. gasseri* G7, *L. plantarum* F1, and *L. helveticus* AC. There were no significant changes of the adhesive properties of *L. delbrueckii* subsp. *bulgaricus* B14 strain after the trypsin treatment. The treatment by metaperiodate, however, significantly decreased the adhesion of *L. delbrueckii* subsp*. bulgaricus* B14 strain, suggesting that cell-bonded exopolysaccharides are involved in the adhesion of this strain.

**Figure 3.**
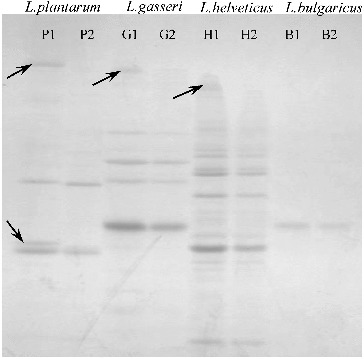
SDS-PAGE of cell-wall proteins of the strains *L. plantarum* F1 (P1 – before, P2 – after adhesion), *L. gasseri* G7 (G1 – before, G2 – after adhesion), *L. helveticus* AC (H1 – before, H2 – after adhesion) and *L. delbrueckii* subsp*. bulgaricus* B14 (B1 – before, B2 – after adhesion).

The cell-wall protein extracts from the strains *L. gasseri* G7, *L. plantarum* F1, *L. helveticus* AC, and *L. delbrueckii* subsp*. bulgaricus* B14 were divided into two parts. The first part (P1, G1, H1 and B1, respectively) was used as a control. The second part (P2, G2, H2 and B2, respectively) was incubated over the monolayers (see the ‘Materials and methods’ section). Figure 3 shows the image after SDS-PAGE of the cell-wall proteins. The protein bands whose relative intensity was markedly decreased after the adhesion are marked with arrows.

Dunne et al. [10] have proved that the pretreatment of *L. salivarius* UCC118 cells with proteolytic enzymes abolished adhesion, indicating the involvement of surface protein(s), such as bacterial adhesin(s). Using SDS-PAGE techniques they determined that the proteolytic treatment resulted in degradation of one cell-wall-associated protein. The present study also demonstrated that the adhesion is mainly due to surface proteins (in three of the four evaluated strains). In the case of the strains *L. gasseri* G7 and *L. helveticus* AC, one cell-wall protein per each strain was demonstrated to be involved in the adhesion, based on the decrease in the relative intensity of the corresponding bands after incubating onto the Caco-2 monolayer.

Surprisingly, however, in the case of *L. plantarum* F1, there was a considerable decrease in the relative intensity of two cell-wall proteins as compared to the control. This could be considered as evidence that two (not one) cell-wall-associated proteins are involved in the adhesion of *L. plantarum* F1, which also proved to be the best adhesive strain in our experiments.

Interestingly, for the strain *L. delbrueckii* subsp*. bulgaricus* B14 we found no change in the cell-wall protein profiles after the adhesion. This could be explained with the fact that cell-bonded exopolysaccharides are most probably the adhesive factors responsible for the adherence of *L. delbrueckii* subsp*. bulgaricus* B14.

The comparative analysis of the adhesive ability of bacterial strains of different species showed the strongest adhesion for the studied *L. plantarum* and *L. gasseri* strains. Strains of these two species with good adhesive properties have also been found in previous studies.[6,14] In our study, two strains of dairy origin, demonstrating relatively good adhesive properties, were observed: *L. delbrueckii* subsp*. bulgaricus* B14 and *L. helveticus* AC. To the best of our knowledge, this is the first description of strains of *L. delbrueckii* subsp*. bulgaricus* and *L. helveticus* with such properties. Strains of *L. delbrueckii* subsp*. bulgaricus* with satisfactory adhesion are of particular interest, as they would allow the development of starter cultures for yogurt with increased probiotic potential.

## Conclusions

In summary, this study provided evidence for the considerable adhesion properties of some *Lactobacillus* spp. strains of intestinal origin and fair adherence of some strains of dairy origin. It is a very rear case to find a *L. delbrueckii* subsp*. bulgaricus* strain with such a degree of adhesion as the one observed in this study. The adhesion of most of the strains was mediated by cell-wall proteins, and in the case of *L. delbrueckii* subsp*. bulgaricus* B14, by cell-wall-bonded exopolysaccharides.
